# Cytotoxic and Antioxidant Properties of Natural Bioactive Monoterpenes Nerol, Estragole, and 3,7-Dimethyl-1-Octanol

**DOI:** 10.1155/2022/8002766

**Published:** 2022-11-23

**Authors:** Mayara Ladeira Coêlho, Muhammad Torequl Islam, George Laylson da Silva Oliveira, Marcus Vinicius Oliveira Barros de Alencar, José Victor de Oliveira Santos, Antonielly Campinho dos Reis, Ana Maria Oliveira Ferreira da Mata, Márcia Fernanda Correia Jardim Paz, Anca Oana Docea, Daniela Calina, Javad Sharifi-Rad, Ana Amélia de Carvalho Melo-Cavalcante

**Affiliations:** ^1^Northeast Biotechnology Network (RENORBIO), Federal University of Piauí, Teresina, Brazil; ^2^Post-Graduation Program in Pharmaceutical Science, Federal University of Piauí, Teresina, Brazil; ^3^Department of Pharmacy, Bangabandhu Sheikh Mujibur Rahman Science and Technology University, Gopalganj 8100, Bangladesh; ^4^Integral Diferencial College (FACID-DEVRY), Teresina, Piaui, Brazil; ^5^Department of Toxicology, University of Medicine and Pharmacy of Craiova, 200349 Craiova, Romania; ^6^Department of Clinical Pharmacy, University of Medicine and Pharmacy of Craiova, 200349 Craiova, Romania; ^7^Facultad de Medicina, Universidad del Azuay, Cuenca, Ecuador, Iran

## Abstract

The therapeutic potential of medicinal plants is noted because of the presence of varieties of biochemicals. The monoterpenes, like nerol, estragole, and 3,7-dimethyl-1-octanol, have been reported for antimicrobial, antifungal, anthelmintic, and antioxidant activities. This study evaluated the toxic, cytotoxic, and oxidant/antioxidant effects of these compounds by several in vitro (DPPH and ABTS radical scavenging, and ferric reducing potential), ex vivo (hemolysis), and in vivo (*Artemia Salina* and *Saccharomyces cerevisiae*) assays. Results suggest that estragole and 3,7-dimethyl-1-octanol at 31.25–500 *μ*g/mL did not exhibit significant cytotoxic effects in the *A. Salina* and hemolysis tests. Nerol showed significant cytotoxic effects on these test systems at all test concentrations. The monoterpenes showed radical (ABTS^•+^ and DPPH^•^) scavenging capacities in a concentration-dependent manner in vitro tests. However, they did not oxidize the genetic material of *S. cerevisiae* (SODWT, Sod1Δ, Sod2Δ, Sod1/Sod2Δ, Cat1Δ, and Cat1Δ/Sod1Δ) lines. Among the three monoterpenes, nerol may be a good candidate for antioxidant and anti-tumor therapies.

## 1. Introduction

**Figure 1:**
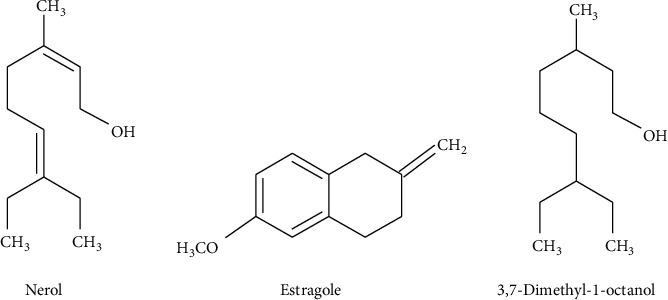
The chemical structure of (a) nerol; (b) estragole, (c) 3,7-dimethyl-1-octanol.

At present, many studies have focused on finding alternatives to traditional treatments for many diseases in plants [1–3]. Active compounds extracted from plants show their efficacy in many pathologies [4–10], even cancers [11, 12], but their application in the clinic is many times limited by low availability in the target organs [13, 14]. The evolution of nanomedicine has tried to solve some of these bioavailability problems of natural bioactive compounds [15], but things are still in the testing step due to the potential toxicity of these nanoformulations [16–19]. Aromatherapy is part of phytotherapy, which uses essential oils extracted from aromatic plants for the treatment or as an adjuvant in the traditional treatment of several clinical pathologies [20–22]. The composition of essential oils is complex and depends on a series of factors, like the species of plant from which they are extracted, the technique used for the extraction, the geographic location where the plant is growing, and also the harvest time [23, 24]. The main components of essential oils are terpenoids and phenylpropanoids [25, 26]. Monoterpenes are chemical compounds found in medicional plant-derived essential oils. They have a pleasant aroma and are used commercially as flavorings, fragrances, and cleaning agents. They are also well-known for their significant biological functions, including their analgesic, antispasmodic, antibacterial, anti-inflammatory, antifungal, and schistosomicidal properties [27]. Nerol, estragole, and 3,7-dimethyl-1-octanol (Figure 1) are monoterpenes that exist in essential oils extracted from various plant species, such as *Croton zehntneri*, *Cymbopogon citrates*, *Cymbopogon nardus*, *Citrus bergamani*, *Zingiber officinalle*, and *Lavandula*sp., and have sedative effects, stimulatory effects on appetite, and beneficial effects on intestinal disorders. They are also known for their attractive, repellent, and toxic effects on insects and microorganisms [10, 28].

Nerol has been noted for its antispasmodic, anthelmintic, antifungal, and antioxidant activities [27, 29]. Estragole was associated with antioxidant and antibacterial activities [30], while 3,7-dimethyl-1-octanol showed antischistosomal [31] and antifungal [32] activity.

Oxidative stress is involved in the onset and/or development of many chronic diseases. Certain defence mechanisms act to maintain the levels of reactive species (e.g., oxygen/nitrogen; ROS/RNS) under normal conditions and to prevent oxidative damage as the action of antioxidant enzymes: superoxide dismutase (SOD), catalase (CAT), glutathione peroxidase (GPx), and glutathione (GSH). Many natural compounds have been investigated for their antioxidant effects through inhibiting free radicals/chemicals that could inhibit oxidative damage [33, 34].

This study evaluated the toxic effects of the monoterpenes nerol (cis-3,7-dimethyl-2,6-octadien-1), estragole, and 3,7-dimethyl-1-octanol in *Artemia salina* and mouse erythrocytes, along with their antioxidant properties in vitro test systems as ABTS^•+^ and DPPH^•^ radical scavenging and ferric reduction potential. Additionally, an in vivo test on *Saccharomyces cerevisiae* strains was also conducted in one wild-type (SODWT), three single-mutant (Sod1Δ, Sod2Δ, and Cat1Δ), and two-double mutant (Sod1/Sod2Δ and Cat1Δ/Sod1Δ) strains.

## 2. Materials and Methods

### 2.1. Reagents and Chemicals

We used 0.05% tween-80 dissolved in isosaline (0.9% NaCl solution) as the vehicle. Trolox (6-hydroxy-2, 5, 7, and 8-tetramethylchroman-2-carboxylic acid) was dissolved in this vehicle. Trolox, potassium dichromate (K_2_Cr_2_O_7_), hydrogen peroxide (H_2_O_2_), Triton X-100, and all the necessary reagents and chemicals, including the test substances (nerol (W277002; purity: ≥97%), estragole (W241105; purity: ≥98%), 3,7-dimethyl-1-octanol (W305774; purity: ≥98%) were obtained from Sigma-Aldrich (St. Louis, MO, USA).

### 2.2. Test Systems

*Artemia salina* and mouse (*Mus musculus*) erythrocytes were used for the cytotoxicity test. 1, 1-diphenyl-2-picrylhidrazyl (DPPH), 2, 2-azobis-(3-ethylbenzothiazoline-6-sulfonate (ABTS), and ferric reduction assays were carried out as in vitro antioxidant capacity test. Six *S. cerevisiae* strains (SODWT, Sod1Δ, Sod2Δ, Cat1Δ, Sod1/Sod2Δ, and Cat1Δ/Sod1Δ) were used for in vivo oxidant and antioxidant assays.

### 2.3. Preparation of Test Compounds

The tested monoterpenes (nerol, estragole, and 3,7-dimethyl-1-octanol) and standards (trolox and K_2_Cr_2_O_7_) were dissolved in the above-mentioned vehicle to obtain the desired concentration for each assay.

### 2.4. *Artemia salina* Bioassay

This test was carried out with a slight modification, as described by Amaral et al. [35]. Briefly, shrimp cysts were incubated in a small aquarium containing a brine solution (50 mg of cysts and 300 mL of brine) for 48 h with direct lighting. Test samples (nerol, 3,7-dimethyl-1-octanol, and estragole) were diluted in the brine solution to get a concentration in the range of 31.25 to 500 *μ*g/mL with a final volume of 5 mL. Ten larvae were added to each tube, and after 24 h, manual observation was done to count the number of live naupli. In the negative (NC) and positive (PC) control groups, saline solution and potassium dichromate (K_2_Cr_2_O_7_) were added, respectively. The concentration range used in the case of the PC group was 31.25 to 500 *μ*g/mL.

### 2.5. Determination of Hemolytic Activity in Mice Erythrocytes

This test was performed on adult Swiss albino mouse (*Mus musculus*) erythrocytes. The mouse was collected from the Central Animal House of the Federal University of Piaui, Brazil. It was allowed free access to standard food and water ad libitum and was kept under controlled lighting (12 h dark/light cycles) at 24 ± 2°C. Blood was collected from the retro-orbital plexus of the mouse (male, 24 gm body weight). Hemolytic activity in mouse erythrocytes was performed, according to Jamialahmadi et al. [36]. Briefly, 300 *μ*L of erythrocyte suspension (10% suspension in PBS, pH 7.4) was mixed with 500 *μ*L of each monoterpene (31.25–500 *μ*g/mL) and placed in a water bath at 37°C for 2 h. After the reaction time, the reaction mixture was centrifuged at 2000 rpm for 5 min, and the absorbance of the supernatant was measured at 540 nm and the results were compared to the triton X-100 (31.25–500 *μ*g/mL).

### 2.6. In Vitro Antioxidant Assays

#### 2.6.1. DPPH^•^ Radical Scavenging Capacity Assay

The DPPH assay was performed according to the method described by Abderrahim et al. [37]. Briefly, a solution containing 300 *μ*L of monoterpenes (0.9–14.4 *μ*g/mL) was added to 2.7 mL of DPPH^•^ solution (0.5 mM in ethanol). After 30 min, the absorbance was measured using a spectrophotometer (Shimadzu, Japan) at 517 nm. A similar concentration of Trolox (TRO) served as the positive control group (PC), while only 0.3 mL of vehicle (0.05% Tween 80 dissolved in 0.9% NaCl solution) was added to the DPPH solution for the negative control (NC) group.

#### 2.6.2. ABTS^•+^ Radical Scavenging Capacity Assay

The test was carried out as per the method of Carvalho [38]. Initially, the ABTS^•+^ was obtained from the reaction of 5 mL of a 7 mM ABTS^•+^ solution with 88 *μ*L of a 2.45 mM potassium persulfate (K_2_S_2_O_8_) solution, which was then incubated at room temperature in the absence of light for 16 h. In the preparation of Trolox (PC), 30 *μ*L of the sample was pipetted and completed with the saline solution. An ABTS solution of 1.960 *μ*L was added to each tube containing monoterpenes (0.9–14.4 *μ*g/mL) and left to stand for six minutes in the dark. The PC group contains the same concentrations of Trolox (as standard), while NC contains 0.05% tween-80 dissolved in a 0.9% NaCl solution (vehicle for monoterpenes). The absorbance was measured using a spectrophotometer at 515 nm.

#### 2.6.3. Ferric Reducing Potential Assay

For this assay, the method described earlier by Singhal et al. [39] was followed. Briefly, a mixture of 500 *μ*L of sodium phosphate buffer (0.2 M, pH 6.6) and 500 *μ*L of 1% potassium ferricyanide was added to the tubes containing monoterpenes (0.9–14.4 *μ*L/mL) and was subsequently placed in a water bath at 50°C for 20 min. After that, 500 *μ*L of 10% trichloroacetic acid, 500 *μ*L of distilled water, and 250 *μ*L of 0.1% ferric chloride were added, followed by an absorbance measurement at 700 nm using a spectrophotometer. The NC and PC groups were carried out with the vehicle (0.05% tween-80 dissolved in 0.9% NaCl solution) and Trolox, respectively.

### 2.7. In Vivo Antioxidant (*S. cerevisiae*) Test

The central disc test was done according to the method presented by Islam et al. [40]. The *S. cerevisiae* strains used in the assay with their genotypes are shown in Table 1. Superoxide dismutase (CuZnSOD-Sod1) is a cytosolic enzyme that is faulty in strain EG118, whereas mitochondrial SOD is faulty in strain EG110 (MnSOD-Sod2). The strain EG133 has both Sod1 and Sod2 defects, while EG223 has a cytosolic catalase deficiency (Cat1). While EG103 is a competent strain that is identical to the wild-type (SODWT), EG is a dual isogenic strain for the Sod1 and Cat1 genes.

### 2.8. Statistical Analysis

The results are expressed as the mean ± standard deviation (SD). The results were evaluated by analysis of variance (ANOVA) followed by a post hoc Newman-Keuls test using GraphPad Prism (version: 6.00; Windows, Graph Pad Software, San Diego California, USA). A *P*-value <0.05 was considered significant criterion.

## 3. Results

### 3.1. Brine Shrimp Lethality Bioassay

**Figure 2:**
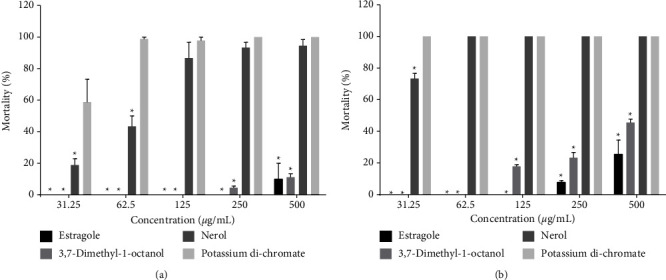
Percentage of *Artemia Salina* mortality under the monoterpene's treatment. Notes: Values are mean ± SD of triplicate value. ^∗^*p*  < 0.05 compared to the NC (vehicle) (ANOVA followed by post-hocNeuman-Keuls test). (a) 24 h and (b) 48 h of treatment with nerol, estragole, 3,7-dimethyl-1-octanol and controls. Vehicles represented 0.0% mortality, therefore, omitted from the figure.

In the brine shrimp lethality bioassay (BSLB), all studied monoterpenes except nerol exhibited low toxicity in 24 hours (Figure 2(a)).

After 48 h of exposure, the nerol showed increased toxicity, more specifically, at the dose of 62.5 *μ*g/mL the mortality was 100% similar to that of the positive control group (Figure 2(b)). Estragole toxicity increased in a dose-dependent manner from the concentration of 250 *μ*g/mL, while 3,7-dimethyl-1-octanol toxicity increased in a dose-dependent manner from the concentration of 125 *μ*g/mL.

### 3.2. Determination of Hemolytic Activity in Mice Erythrocytes

In the hemolysis test, all the monoterpenes showed <25% hemolysis, up to a concentration of 250 *μ*g/mL. However, nerol at 500 *μ*g/mL exhibited hemolysis by 39.12% (Table 2).

### 3.3. In Vitro Antioxidant Assays

**Figure 3:**
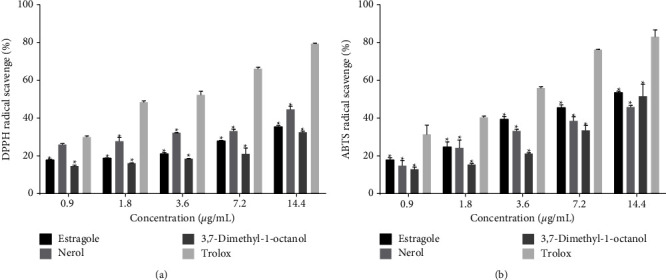
Effects of nerol, estragole, 3,7-dimethyl-1-octanol and Trolox on the inhibition of DPPH (a) and ABTS (b) radicals. Notes: Values are mean ± SD of triplicate value. ^∗^*p*  < 0.05 vs. trolox (ANOVA followed by *t*-Studentpost-hocNeuman-Keuls test). NC showed negligible DPPH (1.23 ± 0.58) and ABTS (1.08 ± 0.23) radical scavenging capacity, therefore, the values have not been shown in the figures.

The radical scavenging capacity of the tested monoterpenes is shown in Figure 3 (DPPH^•^: Figure 3(a) and ABTS^•+^: Figure 3(b)).

**Figure 4:**
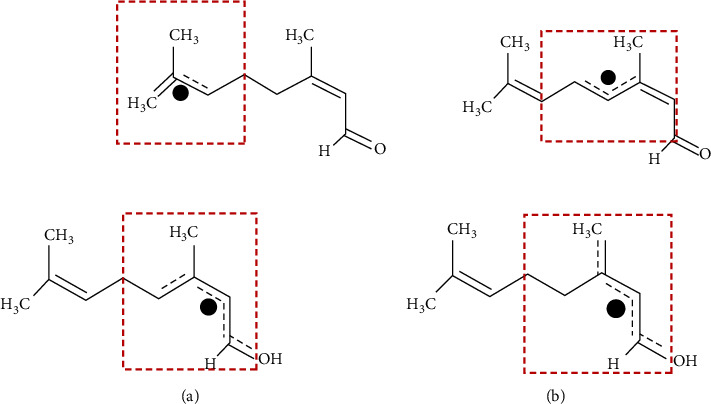
(a) Indication of allylic hydrogens in Nerol. (b) Possible antioxidant mechanism of nerol action in neutralizing R^+^.

The data presented in Figures 3(a) and 3(b) show a variation in the inhibition of DPPH and ABTS radicals in a concentration-dependent manner by the test samples. An increased dose was found to augment an increase in the scavenging capacity of the radicals. Although the activity of the monoterpenes was less than that of the standard drug, Trolox, nerol, and estragole showed the highest scavenging activity against ABTS^•+^ and DPPH^•^, respectively. The presence of allylic hydrogens (number: 13) in nerol (Figure 4(a)), which forms a resonance structure, may be responsible for its antioxidant capacity. Possible hydrogen abstraction and formation of R^+^ is shown in Figure 4(b).

**Figure 5:**
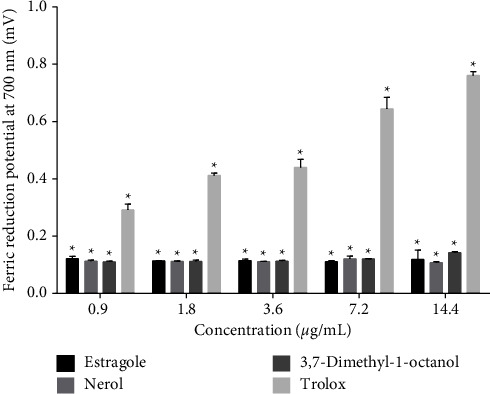
Ferric reduction potential of estragole, nerol, 3,7-dimethyl-1-octanol and Trolox. Notes: Values are mean ± SD of triplicate value. ^∗^*p*  < 0.05 vs. NC (ANOVA followed by *t*-Studentpost-hocNeuman-Keuls test). NC showed negligible ferric reduction capacity (1.33 ± 0.78); therefore, the value has not shown in the figure.

In the ferric reduction assay, all the monoterpenes showed a low reduction capacity of iron (Fe^+3^ to Fe^+2^) compared with the standard drug, Trolox. In this test, all the monoterpenes had almost the same activity at all concentrations tested (Figure 5).

### 3.4. In Vivo Antioxidant (*S. cerevisiae*) Test

Nerol, estragole, and 3,7-dimethyl-1-octanol did not induce oxidative damage at any concentration in any of the tested *S. cerevisiae* strains (Tables 3–5).

**Figure 6:**
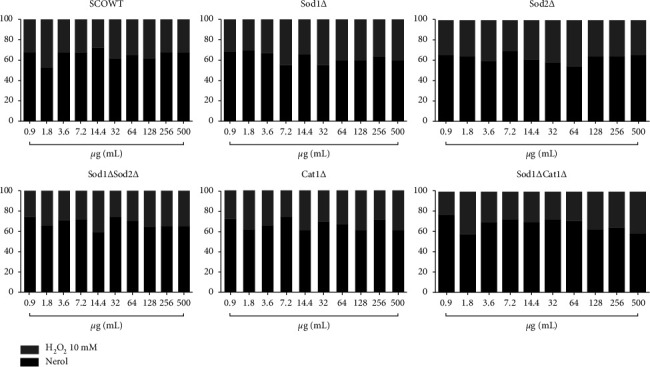
Modulatory effect of nerol in oxidative damage induced by hydrogen peroxide.

**Figure 7:**
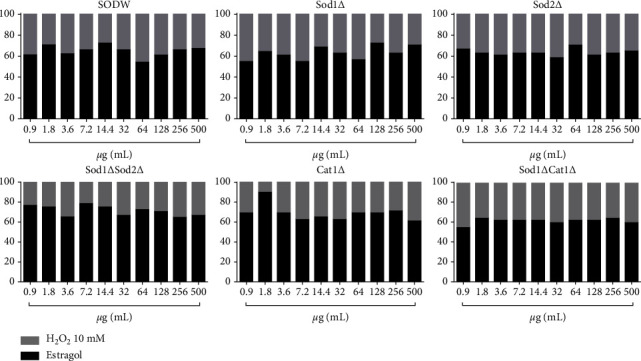
Modulatory effect of estragole in oxidative damage induced by hydrogen peroxide.

**Figure 8:**
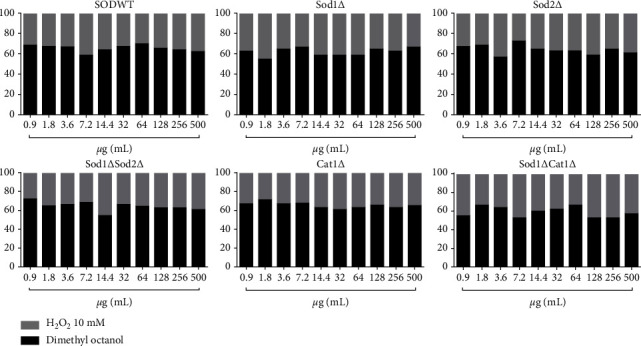
Modulatory effect of 3,7-dimethyl-1-octanol in oxidative damage induced by hydrogen peroxide.

Data indicate that nerol (Figure 6), estragole (Figure 7), and 3,7-dimethyl-1-octanol (Figure 8) showed significant antioxidant activity in *S. cerevisiae*, as they inhibited oxidative damage caused by the stressor, H_2_O_2_ in all strains and concentrations tested. The percentage modulation of oxidative damage was found to be more than 60%.

## 4. Discussion

BSLB is a cheap, rapid, and sensitive test to assay a wide variety of toxicants, including bioactive molecules [42, 43]. In our study, we found a dose-response relationship for all tested monoterpenes along with time-dependent cytotoxicity. The EC_50_ > 1000 *μ*g/mL was classified as inactive (or nontoxic), while the EC_50_ < 100 *μ*g/mL for highly active (very toxic) in the *A. Salina* test [44]. In our study, nerol showed prominent cytotoxicity, which is concurrent with an earlier study [45]. On the other hand, 3,7-dimethyl-1-octanol has been noted for its higher cytotoxic potential compared with estragole.

According to Carvalho [38], toxicity tests should be considered indispensable in the biomonitoring of plant extracts. Another test for toxicological screening of xenobiotics is the in vitro evaluation of hemolytic capacity estimated by erythrocyte damage [46]. There are several variations of this test using human or animal erythrocytes such as rabbits and mice [46–49]. The hemolytic activity in the erythrocytes of mice has a great similarity to the human erythrocytes [44]. This test evaluates the potential of xenobiotics to cause damage to the plasma membrane of the cell, either by the formation of pores or a total rupture of the membrane. This test is very important and can serve as a preliminary test before preclinical and clinical studies [46]. In this study, we observed that of the three tested compounds, nerol has the highest potential to produce hemolysis in a dose-dependent manner, with the highest hemolysis appearing at a concentration of 500 *μ*g/mL.

A persistent radical with a modest rate of deterioration and interactions with the majority of substances, DPPH•. Only powerful reducing chemicals can interact with DPPH to stabilize it in a stoichiometric phase [50, 51]. As opposed to this, 2, 2-azobis-(3-ethylbenzothiazoline-6-sulfonate (ABTS) is oxidized in the presence of K_2_S_2_O_8_ solution and results in the ABTS•^+^ radical. It is very stable and soluble in polar and nonpolar solvents. It remains unaffected by the ionic strength of many solvents. Therefore, this radical is suitable for both polar and nonpolar extracts [44]. In a study, Oliveira [41] also reported a mild antioxidant capacity of estragole at concentrations from 7.83 to 500 *μ*g/mL. In this study, we also observed a concentration-dependent (0.9–14.4 *μ*g/mL) anti-radical activity against DPPH and ABTS radicals. However, the activity was not strong in comparison to the PC group. Estragole showed that scavenger propertied in a dose-dependent manner also in previous studies [30, 52]. In a previous study, we reported the inhibition of lipid peroxidation and protein oxidation by substances having anti-radical and strong reducing capacity [53]. That is why we use the ferric reducing potential technique for evaluating the antioxidant effects of our compounds. The reduction of iron ions (Fe^3+/^Fe^2+^) is influenced by the solubility of the reducing compounds, which in turn is guided by the presence of other acyclic, monocyclic, and bicyclic monoterpene components, derived from phenyl propane and sesquiterpene lactones [44, 54]. Thus, the reducing capacity of the tested monoterpenes may be affected by this consequence. In the case of our compounds, there were no differences between the 3 monoterpenes tested, but all showed low reduction capacity of iron.

*S. cerevisiae* is a popular test system to estimate the oxidative stress capacities of a wider number of biological components. The metabolism of *S. cerevisiae* is quite similar to that of higher eukaryotes, featuring the activation of cytochrome P450 and detoxification [55]. The yeasts, mainly the *S. cerevisiae* species, are widely used in the food and beverage industries and research laboratories [56]. Being a eukaryotic system, *S. cerevisiae* has similarities with mammalian cells at the level of its organelles and macromolecular components, and many of its proteins can be functionally interchangeable with homologous human proteins. Their genetic manipulation is also cheaper compared to other models. In addition, the antioxidant response of *S. cerevisiae* is similar to that of mammalian cells, and 30% of genes related to human diseases are their functional homologues, which makes this test system quite popular [57, 58]. Organisms, in general, are protected against damage caused by oxidative stress by endogenous enzymes such as SOD, CAT, GPx, and glutathione reductase (GR), which can remove free radicals and other oxidizing species and/or repair oxidative damage. These enzymes remove superoxide and peroxides before reacting with catalytic metals to form reactive compounds. However, this inhibition may promote an imbalance in the levels of free radicals, which thus causes DNA damage, protein denaturation, lipid peroxidation, and even cell death.

Medicinal plants rich in monoterpenes are commonly used to treat various diseases. In our study, estragole and 3,7-dimethyl-1-octanol showed low cytotoxicity in *A. salina* and mouse erythrocytes. However, nerol showed high cytotoxic effects at all the doses tested on these test systems. The studied monoterpenes showed a low antioxidant effect in the ferric reducing potential test, but significant inhibition of the oxidative damage caused by H_2_O_2_ in all the *S. cerevisiae* strains was seen at all concentrations of the tested monoterpenes. Regarding the scavenging properties of the monoterpenes, nerol showed a prominent anti-radical capacity in ABTS and DPPH assays, followed by estragole and 3,7-dimethyl-1-octanol.

Antioxidants are of two types, such as primary and secondary. Primary types scavenge free radicals, thereby inhibiting or slowing initiation and/or propagation steps by releasing electrons or hydrogen. This class of antioxidants can react with lipid and peroxyl radicals (e.g., ROO^•^, RO•, and R^•^) and convert them into more stable products (e.g., ROOH, ROH, and RH), for example, phenolic compounds, tocopherols, and synthetic antioxidants (e.g., PG, BHA, BHT, TBHQ). These are also responsible for dimerization processes to terminate the overall reactions. Terpenoids such as monoterpenes have potential antioxidant capacities. These are widely distributed and are able to scavenge almost all types of free radicals [59]. In this study, estragole and 3,7-dimethyl-1-octanol (phenolic monoterpenes) showed promising antioxidant capacity in various test models. Their antioxidant capacity might be responsible for the modulatory effects on hydrogen peroxide-induced oxidative damage in *S. cerevisiae*.

Terpenes, including monoterpenes, play vital roles in plants as they provide defense against various bacteria, fungi, and viruses [60]. These have promising cytotoxic effects against these kinds of pathogens. For example, linalool (a naturally occurring terpene alcohol) is known to exert significant cytotoxic effects by inducing oxidative stress and apoptotic mechanisms [61]. In this study, we also observed that the alcoholic monoterpene nerol exerted better cytotoxic effects on mouse erythrocytes and *S. cerevisiae*.

## 5. Conclusion

The monoterpenes estragole and 3,7-dimethyl-1-octanol did not exhibit significant cytotoxic effects on the test systems (*A. Salina* and mouse erythrocytes). Nerol exhibited significant cytotoxic effects on both test systems in a concentration-dependent manner. All the tested monoterpenes scavenged free radicals' concentration dependently. However, nerol showed a significantly higher antioxidant capacity than estragole and 3,7-dimethyl-1-octanol. The monoterpenes did not oxidize the genetic material of the proficient and deficient *S. cerevisiae* strains. Among the three monoterpenes, nerol may be a good candidate for antioxidant and anti-tumor therapies. Further research is necessary to evaluate the mechanism of action behind the antioxidant and cytotoxic effects in vivo test models.

## Figures and Tables

**Table 1:** *S. cerevisiae* strains used in this study.

Description	Genotype	Manufacturer
EG103 (SODWT)	MATa leu2-3,112 trp1-289 ura 3-52 GAL+	Edith gralla, L angeles
EG118 (Sod1∆)	sod1::URA3 all other markers as EG103	
EG110 (Sod2∆)	sod2::TRP1 all other markers as EG103	
EG133 (Sod1∆Sod2∆)	sod1::URA3 sod2::TRP1 double mutant all other markers as EG103	
EG223 (Cat1∆)	EG103, except cat1:: TRP1	
EG (Sod1∆Cat1∆)	EG103, except sod1:: URA3 and cat1:: TRP1	

The strains were cultured in YEPD medium (Yeast extract 0.5%, bacto-peptone 2% and dextrose 2%) at 28°C HYPERLINK [51]. Suspended cells were seeded in the center of a Petri dish in a continuous cycle. H2O2 (10 mM) was used as an inducer of oxidative stress and formed the positive control (PC) group. Saline 0.9% and DMSO 0.05% groups are the negative control (NC) groups.

**Table 2:** Effects of treatment on hemolytic activity in mouse erythrocytes.

Concentrations (*μ*g/mL)	Hemolysis (%)				
	Estragole	Nerol	3,7-Dimethyl-1-octanol	Triton X-100	NC
31.25	1.67 ± 0.35	2.67 ± 0.35	3.66 ± 0.64	100 ± 0.00^*∗*^	1.23 ± 0.78
62.5	3.49 ± 0.26	4.49 ± 0.26^*∗*^	4.53 ± 0.06^*∗*^	100 ± 0.00^*∗*^	
125	4.45 ± 0.47^*∗*^	5.12 ± 1.05^*∗*^	5.33 ± 0.75^*∗*^	100 ± 0.00^*∗*^	
250	8.21 ± 0.35^*∗*^	6.54 ± 1.78^*∗*^	5.64 ± 0.74^*∗*^	100 ± 0.00^*∗*^	
500	10.28 ± 1.35^*∗*^	34.95 ± 4.74^*∗*^	5.66 ± 0.56^*∗*^	100 ± 0.00^*∗*^	

Values are mean ± SD of triplicate value. ^∗^*p*  < 0.05 vs. NC (ANOVA followed by *t*-Studentpost-hocNeuman-Keuls test).

**Table 3:** Evaluation of oxidative damage by nerol in proficient and deficient strains of *Saccharomyces cerevisiae.*

Treatments	Strains					
	SODWT	Sod1∆	Sod2∆	Sod1∆Sod2∆	Cat1∆	Sod1∆Cat1∆
Saline 0.9%	0.75 ± 0.47	1.75 ± 0.70	0.50 ± 0.28	2.75 ± 1.59	0.50 ± 0.37	1.25 ± 0.50
DMSO 0.05%	0.75 ± 0.47	0.75 ± 0.47	0.9 ± 0.28	0.8 ± 0.32	1.1 ± 0.54	0.5 ± 0.15
H_2_O_2_ 10 mM	30.75 ± 5.0^∗∗^	25.50 ± 4.2^∗∗^	25.25 ± 6.18^∗∗^	27.00 ± 5.09^∗∗^	24.25 ± 4.03^∗∗^	22.25 ± 4.57^∗∗^

Nerol						
0.9 *μ*g/mL	1.50 ± 0.70	1.50 ± 0.70	1.00 ± 0.28	2.00 ± 1.41	1.50 ± 0.70	2.50 ± 2.10
1.8 *μ*g/mL	1.50 ± 0.70	2.00 ± 1.41	3.00 ± 1.41	2.50 ± 0.70	2.50 ± 0.70	2.50 ± 0.70
3.6 mg/mL	1.00 ± 0.28	2.50 ± 0.70	2.50 ± 0.70	2.50 ± 0.70	4.00 ± 1.2	3.00 ± 1.41
7.2 *μ*g/mL	2.00 ± 1.41	3.00 ± 1.41	3.00 ± 1.41	3.00 ± 1.41	2.50 ± 0.70	3.00 ± 1.41
14.4 *μ*g/mL	2.50 ± 0.70	2.50 ± 0.70	3.00 ± 1.41	3.00 ± 0.54	3.00 ± 0.54	3.00 ± 1.41
32 *μ*g/mL	2.00 ± 1.41	3.50 ± 0.70	1.50 ± 0.70	4.50 ± 0.70	3.50 ± 0.70	3.50 ± 0.70
64 *μ*g/mL	2.00 ± 1.41	1.50 ± 0.70	2.50 ± 0.70	2.50 ± 0.70	3.00 ± 1.41	3.00 ± 1.41
128 *μ*g/mL	1.00 ± 0.28	2.00 ± 1.41	2.50 ± 0.70	3.00 ± 1.41	3.00 ± 1.41	2.50 ± 0.70
256 mg/mL	2.00 ± 0.24	2.50 ± 0.70	2.00 ± 1.41	2.50 ± 0.70	1.50 ± 0.70	3.00 ± 1.41
500 mg/mL	1.00 ± 0.28	2.00 ± 0.24	2.00 ± 0.24	2.50 ± 0.70	2.50 ± 0.70	2.50 ± 0.70

*Notes*. Values are mean ± SD. Two-way ANOVA, Tukey post-hoc test. ^∗∗^*p*  < 0.0001 compared to saline solution.

**Table 4:** Evaluation of oxidative damage by estragole in proficient and deficient strains of *Saccharomyces cerevisiae.*

Treatments	Strains					
	SODWT	Sod1∆	Sod2∆	Sod1∆Sod2∆	Cat1∆	Sod1∆Cat1∆
Saline 0.9%	0.75 ± 0.47	1.75 ± 0.70	0.50 ± 0.28	2.75 ± 1.59	0.50 ± 0.37	1.25 ± 0.50
DMSO 0.05%	0.75 ± 0.47	0.75 ± 0.47	0.9 ± 0.28	0.8 ± 0.32	1.1 ± 0.54	0.5 ± 0.15
H_2_O_2_ 10 mM	30.75 ± 5.0^∗∗^	25.50 ± 4.2^∗∗^	25.25 ± 6.18^∗∗^	27.00 ± 5.09^∗∗^	24.25 ± 4.03^∗∗^	22.2 ± 4.57^∗∗^

Estragole						
0.9 *μ*g/mL	2.00 ± 0.54	2.00 ± 1.41	3.00 ± 1.41	3.50 ± 0.70	3.00 ± 1.41	3.50 ± 0.70
1.8 *μ*g/mL	1.50 ± 0.70	1.50 ± 0.70	3.50 ± 0.70	3.50 ± 0.70	4.50 ± 0.70	4.00 ± 2.82
3.6 mg/mL	1.00 ± 1.41	2.50 ± 0.70	2.00 ± 0.54	2.50 ± 0.70	4.00 ± 1.41	4.00 ± 1.41
7.2 *μ*g/mL	2.00 ± 1.41	2.00 ± 1.41	2.00 ± 0.54	2.50 ± 0.70	1.50 ± 2.12	3.50 ± 0.70
14.4 *μ*g/mL	2.50 ± 0.70	2.00 ± 0.54	3.50 ± 0.70	3.50 ± 0.70	4.50 ± 2.12	5.00 ± 0.0
32 *μ*g/mL	2.00 ± 0.54	2.50 ± 0.70	2.00 ± 0.54	2.50 ± 0.70	3.00 ± 1.41	4.50 ± 0.70
64 *μ*g/mL	2.00 ± 0.54	2.50 ± 0.70	3.50 ± 0.70	1.50 ± 0.70	7.00 ± 1.41^∗^	3.00 ± 1.41
128 *μ*g/mL	1.50 ± 0.70	2.50 ± 0.70	3.50 ± 0.70	3.00 ± 1.41	2.50 ± 0.70	4.50 ± 2.12
256 mg/mL	3.50 ± 0.70	2.50 ± 0.70	2.50 ± 0.70	2.50 ± 2.12	3.00 ± 1.41	3.00 ± 2.82
500 mg/mL	1.50 ± 0.70	1.50 ± 0.70	3.00 ± 1.41	2.50 ± 0.70	4.50 ± 0.70	3.50 ± 0.70

*Notes*. Values are mean ± SD. Two-way ANOVA, Tukey post-hoc test. ^∗^*p*  < 0.05 and ^∗∗^*p*  < 0.0001 compared to saline solution.

**Table 5:** Evaluation of oxidative damage by 3,7-dimethyl-1-octanol in proficient and deficient strains of *Saccharomyces cerevisiae.*

Treatments	Strains					
	SODWT	Sod1∆	Sod2∆	Sod1∆Sod2∆	Cat1∆	Sod1∆Cat1∆
Saline 0.9%	0.75 ± 0.47	1.75 ± 0.70	0.50 ± 0.28	2.75 ± 1.59	0.50 ± 0.37	1.25 ± 0.50
DMSO 0.05%	0.75 ± 0.47	0.75 ± 0.47	0.9 ± 0.28	0.8 ± 0.32	1.1 ± 0.54	0.5 ± 0.15
H_2_O_2_ 10 mM	30.75 ± 5.0^∗∗^	25.5 ± 4.2^∗∗^	25.25 ± 6.18^∗∗^	27.00 ± 5.09^∗∗^	24.25 ± 4.03^∗∗^	22.25 ± 4.57^∗∗^

3,7-Dimethyl-1-octanol						
0.9 *μ*g/mL	1.50 ± 0.70	0.50 ± 0.70	1.50 ± 0.70	1.50 ± 0.70	2.50 ± 0.70	2.50 ± 0.70
1.8 *μ*g/mL	1.50 ± 0.70	0.00 ± 0.00	2.00 ± 0.00	1.00 ± 1.41	3.50 ± 0.70	3.00 ± 0.00
3.6 mg/mL	2.00 ± 0.00	1.50 ± 0.70	2.50 ± 0.70	2.00 ± 1.41	2.50 ± 0.70	4.00 ± 1.41
7.2 *μ*g/mL	2.50 ± 0.70	1.00 ± 0.00	2.50 ± 0.70	2.50 ± 0.70	4.00 ± 0.00	3.00 ± 1.41
14.4 *μ*g/mL	2.00 ± 1.41	1.00 ± 1.41	1.50 ± 0.70	2.00 ± 0.00	3.50 ± 0.70	2.00 ± 2.82
32 *μ*g/mL	2.00 ± 0.00	2.50 ± 0.70	3.00 ± 0.00	2.50 ± 0.70	3.50 ± 2.12	3.50 ± 0.70
64 *μ*g/mL	1.50 ± 0.70	3.00 ± 0.00	1.00 ± 0.00	3.50 ± 0.70	3.50 ± 0.70	3.00 ± 1.41
128 *μ*g/mL	1.00 ± 1.41	1.50 ± 0.70	1.50 ± 0.70	3.50 ± 0.70	3.50 ± 2.12	4.00 ± 1.41
256 mg/mL	0.50 ± 0.70	2.50 ± 0.70	1.00 ± 1.41	3.00 ± 1.41	2.50 ± 2.12	3.00 ± 0.00
500 mg/mL	2.50 ± 0.70	3.00 ± 0.00	2.50 ± 0.70	2.50 ± 0.70	3.00 ± 1.41	3.50 ± 0.70

*Notes*. Values are mean ± SD. Two-way ANOVA, Tukey post-hoc test. ^∗∗^*p*  < 0.0001 compared to saline solution.

## Data Availability

The datasets used to support the findings of this study are available from the corresponding authors upon request.
